# Serosurveillance after a COVID‐19 vaccine campaign in a Swiss police cohort

**DOI:** 10.1002/iid3.640

**Published:** 2022-06-06

**Authors:** Parham Sendi, Marc Thierstein, Nadja Widmer, Flora Babongo Bosombo, Annina Elisabeth Büchi, Dominik Güntensperger, Manuel Raphael Blum, Rossella Baldan, Caroline Tinguely, Brigitta Gahl, Dik Heg, Elitza S. Theel, Elie Berbari, Andrea Endimiani, Peter Gowland, Christoph Niederhauser, for the PoliCOV‐19 study

**Affiliations:** ^1^ Institute for Infectious Diseases, University of Bern Bern Switzerland; ^2^ Division Operations Cantonal Police Bern Bern Switzerland; ^3^ Interregional Blood Transfusion Swiss Red Cross Bern Switzerland; ^4^ CTU Bern, University of Bern Bern Switzerland; ^5^ Department of Emergency Medicine Inselspital, Bern University Hospital, University of Bern Bern Switzerland; ^6^ Department of General Internal Medicine Inselspital, Bern University Hospital, University of Bern Bern Switzerland; ^7^ Institute of Primary Health Care (BIHAM), University of Bern Bern Switzerland; ^8^ Division of Clinical Microbiology Mayo Clinic Rochester Minnesota USA; ^9^ Division of Infectious Diseases Mayo Clinic Rochester Minnesota USA

**Keywords:** anti‐NCP‐antibodies, anti‐S‐antibodies, COVID‐19 seroprevalence, SARS‐CoV‐2

## Abstract

**Introduction:**

To assess the risk for COVID‐19 of police officers, we are studying the seroprevalence in a cohort. The baseline cross‐sectional investigation was performed before a vaccination campaign in January/February 2021, and demonstrated a seroprevalence of 12.9%. Here, we demonstrate serosurveillance results after a vaccination campaign.

**Methods:**

The cohort consists of 1022 study participants. The 3‐ and 6‐month follow‐up visits were performed in April/May and September 2021. Data on infection and vaccination rates were obtained via measuring antibodies to the nucleocapsid protein and spike protein and online questionnaires.

**Results:**

The mean age of the population was 41 (*SD* 8.8) years, 72% were male and 76% had no comorbidity. Seroconversion was identified in 1.05% of the study population at the 3‐month visit and in 0.73% at the 6‐month visit, resulting in an infection rate of 1.8% over a time period of 6 months. In comparison, the infection rate in the general population over the same time period was higher (3.18%, *p* = .018). At the 6‐month visit, 77.8% of participants reported being vaccinated once and 70.5% twice; 81% had an anti‐S antibody titer of >250 U/ml and 87.1% of ≥2 U/ml. No significant association between infection and job role within the department, working region, or years of experience in the job was found. Anti‐spike antibody titers of vaccinated study participants showed a calculated decreasing trend 150–200 days after the second vaccine dose.

**Conclusion:**

These data confirm the value of the vaccination campaign in an exposed group other than healthcare professionals.

## Graphical abstract

In this serosurveillance study, we observed a high vaccination and low infection rate during the delta surge in a police cohort. Anti‐Spike antibody titers of vaccinated study participants showed a calculated decreasing trend 150–200 days after the second vaccine dose.

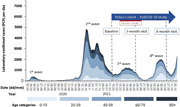

## 1 INTRODUCTION

The COVID‐19 pandemic has ignited social unrest, including domestic violence, a surge in COVID‐19 denials, and antimasking and antivaccine protests worldwide.^1^, ^2^, ^3^ It is reasonable to postulate that police officers, in particular those working in the field, are an exposure population. To assess the risk for SARS‐CoV‐2 infection in this group, since February 2021, we have been studying a cohort of individuals employed by the Cantonal Police Bern in Switzerland.^4^ The seroprevalence of anti‐nucleocapsid antibodies in the police cohort before initiating a vaccine program was 12.9%.^5^ In March 2021, a vaccination campaign for their employees was promoted by the Cantonal Police Bern. Here, we present the COVID‐19 infection and vaccination rate 3 and 6 months after initiating the cohort, and the dynamics of antispike antibody levels in vaccinated individuals. In addition, a comparison between the infection rates in the police cohort and the general population was made to estimate the success of the vaccine campaign.

## 2 METHODS

### 2.1 Cohort

The study protocol is aligned with that of the World Health Organization (WHO) for population‐based age‐stratified seroepidemiological investigations,^6^ adapted for the specific population and geographic region in our study. The population involved in the PoliCOV‐19 study has been published previously,^5^ and included after 6 months 1022 study participants (Appendices, Figure S1).

### 2.2 SARS‐CoV‐2 exposure

**Figure 1:**
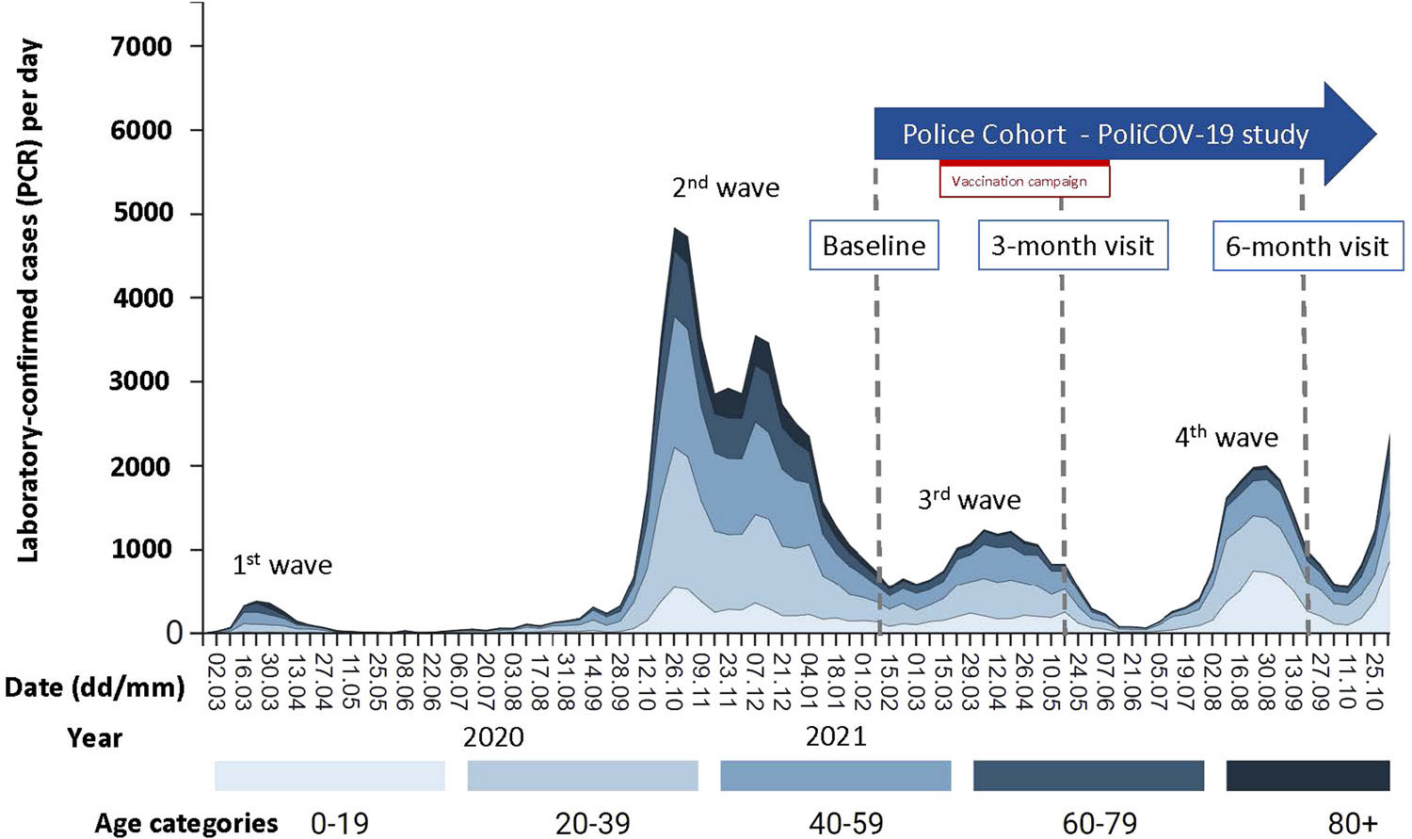
Series of COVID‐19 waves in the canton of Bern (Switzerland) since the onset of the pandemic and the time points of cross‐sectional analysis of the PoliCOV‐19 study. Figure obtained and adapted from open‐source data, available at https://covid-kennzahlen.apps.be.ch/#/de/cockpit (last accessed December 29, 2021).

The series of COVID‐19 waves in our region since the onset of the pandemic and the time points of cross‐sectional analysis are shown in Figure 1. During the study period, there was no government‐ordered lockdown. Wearing face masks for employees of the Cantonal Police Bern was made mandatory during working hours (indoor and outdoor) on October 13, 2020. On June 26, 2021, an exemption was introduced: Wearing face masks was not mandatory for employees of the police within protected indoor rooms of the police departments, under the precondition that a physical distance of 1.5 m was ascertained. For all other circumstances, mask‐wearing remained mandatory.

### 2.3 SARS‐CoV‐2 variants

From mid‐⁠February to the end of June 2021, the SARS‐CoV‐2 Alpha variant (B.1.1.7) was dominant in Switzerland until its replacement by the Delta variant (B.1.617.2, all subvariants AY), which became predominant in late June 2021.^7^

### 2.4 Time points of cross‐sectional analysis

The baseline investigation was performed in January/February and published elsewhere.^5^ The 3‐month follow‐up visit was performed in April/May, and the 6‐month follow‐up in September 2021 (Figure 1).

### 2.5 Questionnaires

During every cross‐sectional analysis of the cohort (i.e., every 3 months), an online questionnaire was sent to study participants. The questionnaire aligned to the survey tools recommended by the WHO,^8^ and a questionnaire used by the Swiss Medical Association (FMH) to evaluate COVID‐19 among physicians in Switzerland,^9^ and then adapted for police officers. It inquired job‐related activity, possible COVID‐19 contact, symptoms consistent with COVID‐19, contact with presumed or confirmed cases, quarantine, and nasopharyngeal test results and vaccination status.

### 2.6 Antibody tests

SARS‐CoV‐2 antibodies to the nucleocapsid protein (NCP) and spike (S) protein were measured by using two commercially available immunoassays (Roche Diagnostics). To increase the specificity of anti‐S antibody test results, we chose a cutoff value of ≥2 U/ml,^10^ instead of ≥0.8 U/ml, as recommended by the manufacturer.

### 2.7 COVID‐19 infection definition in the cohort

COVID‐19 infection was defined as seroconversion of anti‐NCP antibodies or a self‐reported PCR test from a nasopharyngeal swab in the questionnaire. To identify false‐positive serological results, we contacted all individuals with anti‐NCP antibody seroconversion and reinvestigated the cases. Samples with low titer results from individuals with no symptoms or negative nasopharyngeal PCR test results were reanalyzed with a second and different anti‐NCP antibody assay (Bio‐Rad). In the case of seronegative results with the second assay, the serum test result was considered as a possible or likely false‐positive result. Serological results from individuals with a self‐reported positive nasopharyngeal PCR test result, with symptoms but without anti‐NCP seroconversion were considered as possible or likely false‐negative cases, if the time interval between PCR test result and serum sampling was ≥14 days.

### 2.8 The infection rate in the general population

New infection cases in the general population are defined as laboratory‐confirmed cases (positive PCR test from nasopharyngeal or saliva sample). The data were obtained from the Federal Office of Public Health.^11^ The canton of Bern consists of more than 1,043,000 inhabitants; the age‐matched population for this study consisted of 671,678 registered inhabitants at the 3 month‐visit and 669,243 at the 6‐month visit.

### 2.9 COVID‐19 vaccine

The messenger RNA vaccines from Pfizer‐BioNTech and Moderna are authorized and approved for use in Switzerland. The vaccination campaign of the police was promoted from March 12th till June 11th, 2021 (Figure 1).

### 2.10 Primary endpoint

The primary endpoint was the infection rate in the police cohort at the 3‐ and 6‐month visit.

### 2.11 Secondary endpoints

The secondary endpoints included the comparisons of the infection rates between the police cohort and the general population at the 3‐ and 6‐month visits, the association of age, comorbidity, job role (i.e., mainly fieldwork or mainly office work), working department, working region, and years of experience with the infection rate. Secondary endpoints included further the proportions of vaccinated individuals and those with anti‐S antibody titers ≥2 U/ml in the cohort at the 6‐month visit. In vaccinated individuals, the time interval from vaccination to the calculated trend of anti‐S antibody titers falling below 250 U/ml was defined as a secondary endpoint, also.

### 2.12 Statistical analysis

To describe the characteristics of the study cohort, we used mean ± standard deviation (*SD*) or median with interquartile range for summarizing continuous variables, as appropriate. Comparisons were made by using the Student *t* test or Mann–Whitney test, respectively. Categorical data were shown as numbers with percentages and compared by using Fisher's exact test for binary variables or the Chi‐squared test for more than two categories. The Chi‐squared test of homogeneity was used to compare new infection rates between the police cohort and the Bernese general population (binary variables). The comparisons included both the overall infection rates and were matched by age groups according to the following categories: 20–29, 30–39, 40–49, 50–59, and 60–69 years. For comparative analysis to identify groups at risk for infection, the variables comorbidity, working department, and working region were combined with age groups and years of experience within the police department. The latter was categorized as 0–9, 10–19, 20–29, and 30 or more years of experience.

Generalized additive models were used to estimate the trend of the anti‐S antibody titers over time after vaccination. All analyses were performed with R (version 3.6.2).

## 3 RESULTS

The mean age of the 1022 study participants was 41 (*SD* 8.8) years, 72% were male and 76% had no comorbidity; 58.3% (560) of study participants indicated that their main activity was fieldwork. The numbers of samples analyzed at baseline, 3‐month visit and 6‐month visit were 978, 997, and 982, respectively. The presence or absence of seroconversion between the baseline and 3‐month visit was investigated in 956 paired samples, and between the 3‐ and 6‐month visits in 955 paired samples. The seroprevalences of anti‐NCP antibodies—without adjusting for paired sample results or false positive or false negative results—were 12.9% at baseline,^5^ 14.4% at the 3‐month visit, and 15.3% at the 6‐month visit (Appendices, Figure S2).

### 3.1 Primary endpoint—COVID‐19 infection in the cohort

Seroconversion was identified in 1.05% (10/956) at the 3‐month visit (Table 1A) and in 1.15% (11/955) at the 6‐month visit (Table 1B). At the 3‐month visit, no false‐positive results were detected; at the 6‐month‐visit, 4 of the 11 positive results were likely false‐positive serological test results (Table 1B). Therefore, the proportion of individuals with seroconversion at 6 months was adjusted from 1.15% (11/955) to 0.73% (7/955). The seroprevalence after excluding nonpaired samples was adjusted to 13.95% at the 3‐month‐visit, and 14.7% at the 6‐month visit.

**Table 1A:** Newly identified COVID‐19 cases between baseline (February/March 2021) and the 3‐month visit (April/May 2021)

3‐month visit: 10 (1.05%) newly identified COVID‐19 infections in 956 study participants with samples at baseline and 3‐month visits								
Record ID	Symptoms consistent with COVID‐19	NSP swab	Seroconversion Dates of sampling^a^	Anti‐NCP (COI)^b^	Anti‐S (U/ml)^b^	Vaccinated	1st Dose	2nd Dose
43^c^	Yes (Onset: April 10)	Negative	March 4/May 4	176	>250	Yes	March 26	May 4
118	Yes (Onset: February 14)	Negative	March 4/May 4	45.1	>250	Yes	April 10	May 10
123	No, and no known or traceable contacts	Not tested	Feb 16/May 4	104	>250	Yes	March 10	May 14
130	No	Positive March 20	Feb 16/May 11	106	104	No	–	–
291	Yes	Positive April 21	Feb 26/June 11	123	154	No	–	–
702	Yes	Positive February 27	March 9/April 27	4.2	74.4	No	–	–
739	Yes (Onset: March 22)	Negative	Feb 23/April 26	93.2	25.7	No	–	–
771	Yes (Onset: End of February)	Negative	March 4/May 12	12.5	>250	Yes	May 5	–
813	Yes	Positive March 30	Feb 25/April 26	135	>250	No	–	–
979	Yes	Positive April 12	March 4/April 27	128	18.1	No	–	–

*Note*: PCR in case of a positive result. Antigen test or PCR in case of a negative result. Self‐reported results in questionnaires.

Abbreviations: Anti‐NCP, antinucleocapsid antibodies; Anti‐S, antispike protein antibodies; COI, cut‐off index; NSP, nasopharyngeal swab testing.

^a^ The first date is the date of sampling at baseline (seronegative); the second date is the date of sampling at the 3‐month visit (seropositive).

^b^ Results at the 3‐month visit; results at baseline are not shown because they are seronegative.

^c^ COVID‐19 disease between the first and second vaccination dose.

**Table 1B:** Newly identified COVID‐19 cases between the 3‐month (April/May) and the 6‐month visit (September 2021)

6‐month visit: 11 (1.15%) newly identified COVID‐19 infections in 955 study participants with samples at 3‐month and 6‐month visits. Four results are possibly or likely false positive (i.e., 7 [0.73%] newly identified COVID‐19 infections)								
Record ID	Symptoms consistent with COVID‐19	NSP swab	Seroconversion Dates of sampling^a^	Anti‐NCP (COI)^b^	Anti‐S (U/ml)	Vaccinated	1st Dose	2nd Dose
31^c^	Yes (Onset: April 22)	Negative	April 27/Sept 8	16.4	>250	Yes	March 29	April 30
195	No, and no known or traceable contacts	Not tested	April 27/Sept 8	14.4	>250	Yes	April 27	May 25
220^d^	Yes (Onset: August 27)	Negative	May 7/Sept 8	2.1^d^	>250	Yes	April 19	May 17
245^d^	No, and no known or traceable contacts	Not tested	April 28/Sept 10	1.0^d^	0.4	No	–	–
322^d^	No, and no known or traceable contacts	Not tested	April 27/Sept 9	1.3^d^	>250	Yes	April 27	May 24
380^c^	Yes (Onset: Mid‐May)	Positive May 10	May 7/Sept 20	29.2	>250	Yes	April 20	Sept 27
465	No, and no known or traceable contacts	Not tested	April 30/Sept 9	23.7	>250	Yes	April 20	May 20
610	Yes	Positive August 21	May 6/Oct 26	135	30	No	–	–
717	Yes (Onset: September)	Negative	April 26/Oct 29	23.2	>250	No	–	–
841	Yes	Positive Sept 14	April 9/Sept 27	11.2	>250	Yes	April 9	May 27
933^d^	No, and no known or traceable contacts	Not tested	April 26/Sept 21	3.3^d^	>250	Yes	April 23	May 21

*Note*: PCR in case of a positive result. Antigen test or PCR in case of a negative result: Self‐reported results in questionnaires.

Abbreviations: Anti‐NCP, antinucleocapsid antibodies; Anti‐S, antispike protein antibodies; COI, cut‐off index; NSP, nasopharyngeal swab testing.

^a^ The first date is the date of sampling at the 3‐month visit (seronegative); the second date is the date of sampling at the 6‐month visit (seropositive).

^b^ Results at the 6‐month visit; results at the 3‐month visit are not shown because they are seronegative.

^c^ COVID‐19 disease between the first and second vaccination dose.

^d^ False‐positive anti‐NCP result possible or likely

No breakthrough infections were seen after two doses of vaccination. Six of 10 infections at the 3‐month visit (Table 1A), and three infections at the 6‐month visit occurred in non‐vaccinated individuals (Table 1B). The remaining infections occurred in vaccinated individuals between the first and the second dose of the vaccine.

### 3.2 Secondary endpoints

COVID‐19 infection proportions in the cohort in comparison to the ones in the general population:In the police cohort, the increase in seroprevalence at the 6‐month visit was 1.80% in comparison to the baseline (i.e., from 12.9% to 14.7%), and 0.73% in comparison to the 3‐month visit (i.e., from 13.95% to 14.7%). These values were significantly lower in comparison to the increase of the calculated infection rate of the general population (in comparison to the 6‐month span: 1.8% vs. 3.18%, *p* = .018; in comparison to 3‐month span: 0.73% vs. 1.77%, *p* = .021). After matching for age groups, the infection rate was lower in the police cohort than that in the general population, though not statistically significant (Appendices, Figures S3–1 and S3–2).

No statistically significant difference was seen in the subgroup analysis when comparing police officers involved in the fieldwork activity and the age‐matched general population. No statistically significant association was found in the comparative analysis, including comorbidity, job role within the department, and years of experience (Appendices, Table S1).

### 3.3 Vaccination rate and antispike antibody titers in the cohort

**Figure 2:**
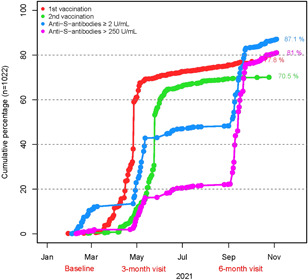
Cumulative proportion of vaccinated individuals in the police cohort. who were vaccinated or recovered from COVID‐19. The proportion of individuals with anti‐S antibody titers >250 U/ml likely represented the vaccinated group because it included responders and nonresponders of questionnaires. The group with anti‐S antibody titers of ≥2 U/ml consisted of the proportions of both, individuals who were vaccinated and those who had recovered from COVID‐19 irrespective of vaccination status. The timeline is biased by the predefined time point of serum sampling and filling out questionnaires.

At the 6‐month visit, 77.8% of participants reported being vaccinated once and 70.5% twice; 81% had an anti‐S antibody titer of >250 U/ml and 87.1% of ≥2 U/ml (Figure 2). The proportion of individuals with anti‐S antibody titers >250 U/ml likely represented most of the the vaccinated group because it included responders and nonresponders of questionnaires. The proportions of these parameters among police officers mainly involved in fieldwork and those mainly involved in office work were similar; 85.0% and 84.5% (*p* = .9), respectively, reported being vaccinated once, 79.3% and 79.4%% (*p* = 1.0) reported being vaccinated twice, 84.6% and 83.6% (*p* = .748) had an anti‐S antibody titer of >250 U/ml, and 90.2% and 88.1% (*p* = .377), respectively, had an anti‐S antibody titer ≥2 U/ml. The group with anti‐S antibody titers of ≥2 U/ml consisted of the proportions of both, individuals who were vaccinated and those who had recovered from COVID‐19, irrespective of vaccination status.

A total of 2 (0.3%) double‐vaccinated and immunocompromised individuals did not show anti‐S antibodies at the time of point serum sampling. A total of 56 (5.7%) of the study participants were seropositive and reported not being vaccinated.

### 3.4 Dynamics of anti‐S antibody levels

**Figure 3:**
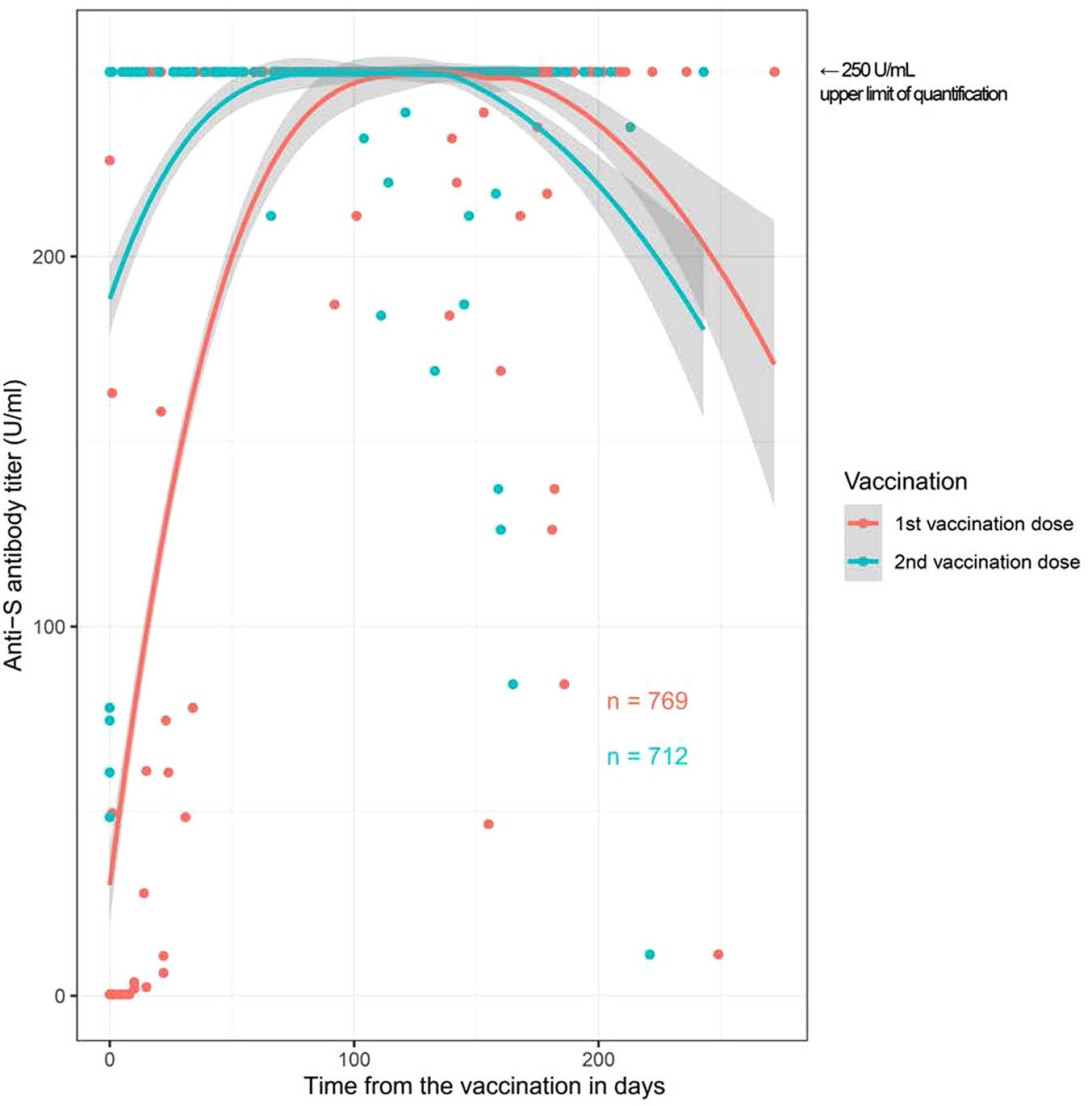
Calculated trend of anti‐S antibody titer curve over time in vaccinated study participants. Each dot reflects the sampling time point. The dynamics of antibody titers over time are biased by the predefined serum sampling time points.

Anti‐S antibody titers of vaccinated study participants showed a calculated decreasing trend after 150–200 days (Figure 3).

## 4 DISCUSSION

In this cohort study, we noted a low infection rate and a relatively high vaccination rate among police officers. Despite a presumed higher exposure to SARS‐CoV‐2, in particular for police officers mainly involved in the fieldwork activity, the overall infection rate was not higher than in the general population. Finally, 150–200 days after vaccination, a decreasing trend in anti‐S antibody titers was observed, underscoring the necessity of a booster vaccine 4–6 months after the second dose.

Law enforcement personnel face physical and psychological challenges during the COVID‐19 pandemic.^12^, ^13^ Their exposure to SARS‐CoV‐2 and possible risk of transmission during working hours (e.g., in attendance of public protests) have been scarcely investigated. Seroprevalence studies are useful means to estimate the true extent of SARS‐CoV‐2 infection among a population.^14^, ^15^ Few seroprevalence studies focused on public safety personnel.^16^, ^17^, ^18^, ^19^, ^20^, ^21^, ^22^ Garbarino et al.^20^ reported an overall seroprevalence of 4.8% in 10,535 police officers in Italy, with a higher seroprevalence in northern (9%) than in southern regions (1.6%).

In our cohort, the seroprevalence at the baseline was 12.9% in February 2021, similar to that reported in the general population.^5^ The self‐reported compliance with mask‐wearing during working hours was very high. The results suggested that household contacts were the leading transmission venues. Regional differences in the seroprevalence were observed, and police officers mainly working in the field were more frequently seropositive than those mainly working in the office.^5^ In this study, the cohort was followed for 6 months. The regional and job‐related differences in seroprevalence within the cohort waned over this time period. However, at the 6‐month visit, the infection rate was lower in the cohort than the one in the general population (1.8% vs. 3.18%, *p* = .018). The true difference was likely more pronounced, considering that the observation in the police cohort was more precise than the one in the general population and that the numbers in the general population are likely underestimated. Although the difference in the proportion of the infection rate between the police cohort and the general population was minor, the calculated absolute number of individuals in the entire population is considerable.

In our view, the differences in COVID‐19 infection rates are likely explained by the high compliance of police officers with hygiene precautions and mask‐wearing with contacts, and by the relatively high vaccination rate. Previous studies have shown the efficacy of COVID‐19 messenger RNA vaccines.^23^ McLaughlin et al.^24^ calculated in a negative binomial regression model that US counties with ≥80% of vaccine‐eligible persons fully vaccinated had 30% lower rates of COVID‐19 cases and 46% lower rates of COVID‐19‐related deaths compared to US counties with <50% vaccine coverage. The vaccination rate in the police cohort was likely more than 80% when considering the responses in the questionnaires and the proportions of individuals with anti‐S antibody titers of >250 U/ml. A high proportion of study participants were vaccinated before the 4th wave of the pandemic. This proportion was higher than the one reported for the general population during the same time period. For comparison, 54%–64% of the general population received at least 1 dose, and 58%–59% received 2 doses of COVID‐19 vaccine in Switzerland in September 2021.^11^ These proportions include elderly individuals who were prioritized in the vaccine distribution. Thus, the age‐matched differences in the vaccine rate between the police cohort and the general population were likely higher because retired individuals were not included in the police cohort while they were included in the vaccination registry of the general population. The lower proportion of vaccination in the general population cannot be explained by the accessibility to the vaccine. In Switzerland, the priority for receiving a vaccine depended on the risk of a severe course of COVID‐19 and immune status. However, during the study period, all individuals had access to a COVID‐19 vaccine. Because vaccination is not mandatory, individuals who are skeptical about the COVID‐19 vaccine can refuse to be vaccinated.

The overall proportion of individuals with antibodies against SARS‐CoV‐2 in the police cohort—defined as the proportion of individuals with anti‐S antibody titer ≥2 U/ml—was 87.1% in September 2021. Similar to other studies,^25^ we observed a waning humoral response after vaccination. In our previous baseline study,^5^ we demonstrated that the neutralization capacity of naturally acquired antibodies decreased with emerging of new variants of SARS‐CoV‐2, and that neutralization correlated with the extent of antibody titer. Vaccine efficacy decreases over time.^26^, ^27^ A vaccine booster dose increases the antibody neutralization level and leads to increased protection against infection of the delta variant and severe illness.^28^, ^29^ However, this effect is likely not durable. In the police cohort, the calculated population curve of the sample results indicated a decrease in anti‐S antibody titers below 250 U/ml approximately 150–200 days after vaccination. The aforementioned arguments together with these results justified promoting a booster vaccination (third dose) campaign.

Our study has limitations. The statistically significant difference in infection rate between the police cohort and the general population is arguable because it was only seen in the overall analysis but not in the age‐matched comparison. We were unable to exclude the infection rate in risk groups within the general population, considering that the police cohort consists of predominantly healthy individuals. However, the true infection rate in the general population is likely underestimated. We were unable to identify an infection in individuals without self‐reported nasopharyngeal sample test results and at least two serum (paired) samples over the 6 months (i.e., to detect seroconversion). We believe that our results are representative, considering that in more than 95% of study participants two or three serum samples were available. The time points for blood sampling and sending out questionnaires were predefined in the study protocol. Hence, the dynamics of antibody titers over time are biased by these sampling time points. The COVID‐19 infection rate in the police cohort was evaluated by self‐reported PCR test results and seroconversion in serum samples. The infection rate in the general population was evaluated by analyzing laboratory‐confirmed cases that were reported daily by the Federal Office of Public Health. Despite using two different methodologies, we were able to statistically homogenize these results for comparison. We are unable to perform antibody titer dynamic analysis at very high titer levels, because of the upper quantification limit of the anti‐S antibody assay (i.e., >250 U/ml).

In conclusion, our COVID‐19 cross‐sectional surveys among police officers demonstrated an increase in seroprevalence from 12.9% to 14.7% in 6 months. The increase was lower than the laboratory confirmed SARS‐CoV‐2 infection rate observed in the general population. During the same period, we observed a relatively high vaccination rate of approximately 80%. In contrast to the pre‐vaccination analysis at baseline, no significant association with the job role within the department or working regions was observed. The observed waning humoral response 150–200 days after vaccination together with results from other studies showing the efficacy of a third dose, supported a further campaign for a booster vaccination. The results of the cross‐sectional surveys at the 9‐ and 12‐month visit are currently being analyzed.

## CONFLICTS OF INTEREST

The authors declare no conflicts of interest.

## AUTHOR CONTRIBUTIONS

**Parham Sendi, Marc Thierstein, Rossella Baldan, and Christoph Niederhauser** designed the study, were responsible for the conduction of the study, and **Parham Sendi** wrote the first draft of the manuscripts. **Nadja Widmer, Peter Gowland, Caroline Tinguely, and Christoph Niederhauser** were responsible for performing the ECLIA assays and data transfer. **Annina Elisabeth Büchi** was responsible for data monitoring. **Flora Babongo Bosombo, Brigitta Gahl, and Dik Heg** performed the statistical analysis. **Dominik Güntensperger** was responsible for data management. **Elitza S. Theel, Elie Berbari, and Andrea Endimiani** contributed to the study design and research analysis and provided their scientific expertise for this study. All authors revised the first draft, read and approved the final manuscript.

## ETHICS STATEMENT

The study was performed in accordance with the Helsinki Declaration of 1964, and its later amendments. The collection of coded data and the design of the work were approved by the Cantonal Research Ethics Commission of Bern, Switzerland (ID‐2020‐02650). **Trial registration**: ClinicalTrials.gov NCT04643444. All participants signed written informed consent before enrolment in the PoliCOV‐19 study.

## Supporting information

Supporting information.

## Data Availability

Questionnaires can be requested from the corresponding author. Data sharing will be considered under the form of collaborative projects. Proposals can be directed to the corresponding author.
