# Supplementary material for: Serosurveillance after a COVID‐19 vaccine campaign in a Swiss police cohort
Source: Immun Inflamm Dis. 2022 Jun 6;10(7):e640. doi: 10.1002/iid3.640 (PMC9168549; doi:10.1002/iid3.640)
Supplement: Supplementary file 1 — Supporting information. [file IID3-10-0-s001.docx]

**SUPPLEMENTARY MATERIAL to Original Article**

**High Vaccination and Low SARS-CoV-2 Infection Rate in a Swiss Police Cohort During the Delta Wave.**

Parham Sendi,^1*^ Marc Thierstein,^2^ Nadja Widmer,^3^ Flora Babongo Bosombo,^4^ Annina Elisabeth Büchi,^5^ Dominik Güntensperger,^4^ Manuel Raphael Blum,^6,7^ Rossella Baldan,^1^ Caroline Tinguely,^3^ Brigitta Gahl,^4^ Dik Heg,^4^ Elitza S. Theel,^8^ Elie Berbari,^9^ Andrea Endimiani,^1^ Peter Gowland,^3^ Christoph Niederhauser^1,3^ for the PoliCOV-19 study.

^1^Institute for Infectious Diseases, University of Bern, Bern, Switzerland.

^2^Division Operations, Cantonal Police Bern, Bern, Switzerland.

^3^Interregional Blood Transfusion Swiss Red Cross, Bern, Switzerland.

^4^CTU Bern, University of Bern, Bern, Switzerland.

^5^Department of Emergency Medicine, Inselspital, Bern University Hospital, University of Bern, Bern, Switzerland.

^6^Department of General Internal Medicine, Inselspital, Bern University Hospital, University of Bern, Bern, Switzerland.

^7^Institute of Primary Health Care (BIHAM), University of Bern, Bern, Switzerland.

**^8^**Division of Clinical Microbiology, Mayo Clinic, Rochester, Minnesota, USA.

**^9^**Division of Infectious Disease, Mayo Clinic, Rochester, MN, USA.

**Keywords**: SARS-CoV-2; anti-S-antibodies; anti-NCP-antibodies; COVID-19 seroprevalence.

***Correspondence:** Parham Sendi, MD, ORCID: 0000-0002-7347-6312

Institute for Infectious Diseases, University of Bern, Friedbühlstrasse 51, 3010, Bern, Switzerland.

[parham.sendi@ifik.unibe.ch](mailto:parham.sendi@ifik.unibe.ch)

Tel: +41 31 638 69 86; Fax: +41 31 638 67 86

**Content**

Page 3: **Appendices Figure S1:** The population involved in the PoliCOV-19 study has been published previously (Open Forum Infect Dis. 2021 Oct 16;8(12):ofab524. doi: 10.1093/ofid/ofab524), and included after 6 months 1022 study participants

Page 4: **Appendices Figure S2:** Anti-NCP antibody seroprevalence of the police cohort at baseline and 3- and 6-month visits, without correcting for paired samples, false positive or false negative results.

Page 5: **Appendices Figure S3-1:** Comparison of the SARS-CoV-2 infection rate between the police cohort and the general population of the canton of Bern. Comparisons between the infection rates at the 3-month and 6-month visits.

Page 6: **Appendices Figure S3-2:** Comparison of the SARS-CoV-2 infection rate between the police cohort and the general population of the canton of Bern over a time period of 6 months (i.e., from February to September 2021).

Page 7: **Supplementary Table S1:** Association of comorbidity and work-related factors with the infection rate:


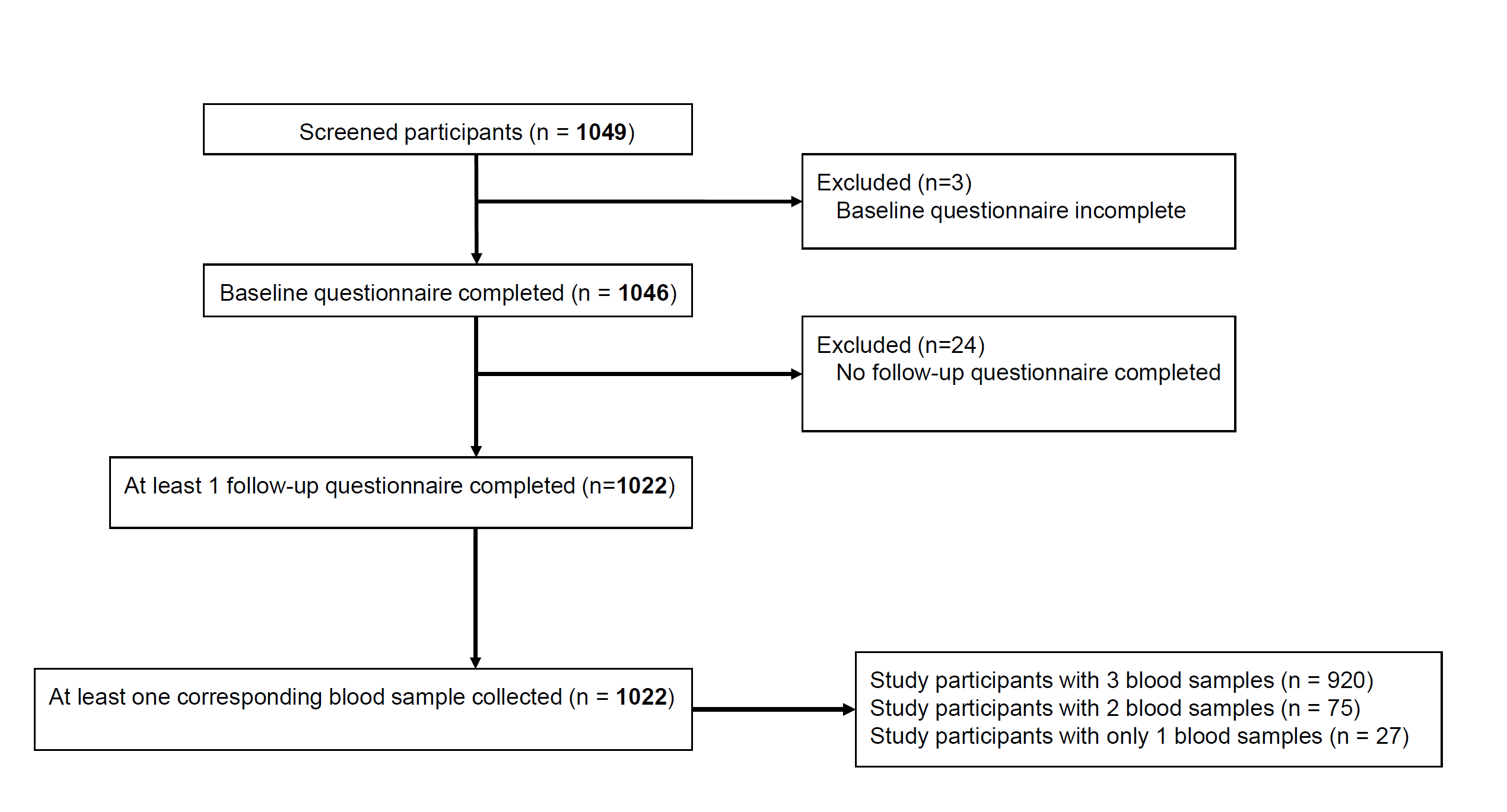


**Figure S1:** Number of individuals included in the cohort analysis between January/February and September 2021 **(n = 1022)**


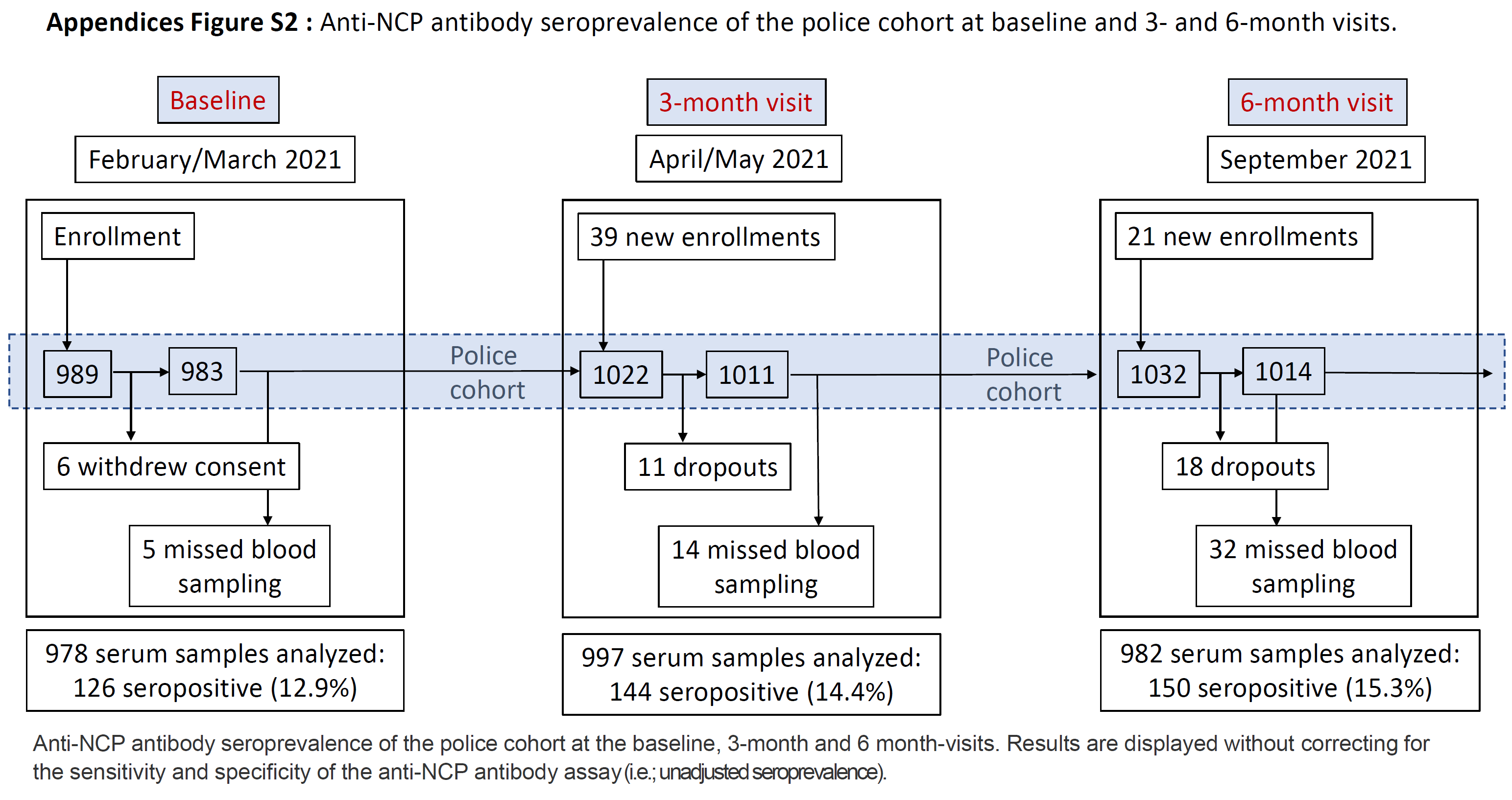

**Appendices Figure S3-1**: Comparison of the SARS-CoV-2 infection rate between the police cohort and the general population of the canton of Bern. Comparisons between the infection rates at the 3-month (April/May 2021) and 6-month visits (September 2021).

**Appendices Figure S3-2**: Comparison of the SARS-CoV-2 infection rate between the police cohort and the general population of the canton of Bern over a time period of 6 months (i.e., from February to September 2021).

**Appendices Table S1: Association of comorbidity and work-related factors with the infection rate:**

|  | **Comorbidity** | | | | | |
| --- | --- | --- | --- | --- | --- | --- |
|  | **Yes** | | | **No** | | |
| **Age group** | beta | Odds ratio | p-value | beta | Odds ratio | p-value |
| **20 - 29** | ref |  |  | ref |  |  |
| **30 - 39** | 17.168 | 28578730 | 0.999 | 0.025 | 1.025 | 0.938 |
| **40 - 49** | 0 | 1 | 1 | -0.161 | 0.851 | 0.629 |
| **50 - 59** | 18.131 | 74849054 | 0.999 | -0.092 | 0.912 | 0.805 |
| **60 - 69** | 0 | 1 | 1 | -0.297 | 0.743 | 0.788 |
| **Intercept** | -19.566 | 0 | 0.999 | -1.649 | 0.192 | <0.001 |
|  | **Department** | | | | | |
|  | **Regional police** | | | **Others** | | |
| **Years of experience** | beta | Odds ratio | p-value | beta | Odds ratio | p-value |
| **0 - 9** | ref |  |  | ref |  |  |
| **10 - 19** | -0.266 | 0.766 | 0.285 | 0.491 | 1.634 | 0.297 |
| **20 - 29** | 0.144 | 1.155 | 0.653 | 0.498 | 1.645 | 0.32 |
| **> 30** | 0.175 | 1.191 | 0.739 | -0.954 | 0.385 | 0.384 |
| **Intercept** | -1.561 | 0.21 | <0.001 | -2.181 | 0.113 | <0.001 |
|  | **Work region** | | | | | |
|  | **Bern City, Region Bern** | | | **Bernese Oberland; Mittelland, Emmental, Oberaargau; Seeland, Bernese Jura** | | |
| **Years of experience** | beta | Odds ratio | p-value | beta | Odds ratio | p-value |
| **0 - 9** | ref |  |  | ref |  |  |
| **10 - 19** | -0.037 | 0.963 | 0.912 | -0.167 | 0.846 | 0.541 |
| **20 - 29** | 0.229 | 1.257 | 0.54 | 0.094 | 1.098 | 0.791 |
| **> 30** | -0.436 | 0.647 | 0.576 | -0.196 | 0.822 | 0.734 |
| **Intercept** | -1.915 | 0.147 | <0.001 | -1.462 | 0.232 | <0.001 |
